# Validation of Herek’s attitudes toward lesbian women and gay men scale among undergraduates in mainland China

**DOI:** 10.3389/fpsyg.2022.842397

**Published:** 2022-10-06

**Authors:** Junfang Wang, Yusi Liu, Guochen Fu, Yifan Chen, Lei Wu, Mingliang Pan, Yuli Yang, Zhuo Chen, Yu Cao, Yong Li, Hao Wang, Bixiang Wang, Ruyi Du, Yanting Xiong, Wei Liu, Nuo Xu, Xiaobao Xia, Qianqian Li, Chengcheng Lv, Fang Ruan

**Affiliations:** ^1^Department of Preventive Medicine, Hubei University of Science and Technology, Xianning, China; ^2^National Demonstration Center for Experimental General Medicine Education of Hubei University of Science and Technology, Xianning, China

**Keywords:** ATLG scale, confirmatory factor analyses (CFA), undergraduates, mainland China (PRC), reliability

## Abstract

The lack of a standardized reliable and valid instrument makes it difficult to measure attitudes toward lesbian women and gay men (ATLG) consistently and thus poses a challenge to compare and contrast intervention measures. This study aimed to validate Herek’s ATLG scale among undergraduates in mainland China and identify factors associated with negative attitudes toward LG. A total of 6,036 eligible undergraduates conveniently drawn from 30 provinces across mainland China were randomly split in half. Item analysis was first used to select unrelated or redundant items for deletion. Exploratory factor analysis (EFA) were then conducted on the first half of the sample (*n* = 3,001), followed by confirmatory factor analysis (CFA) and reliability analysis in the second half (*n* = 3035). Logistic regression analyses were finally carried out to identify their determinants. Six items were removed from the item analysis. EFA supported the existence of two factors (ATL and ATG). CFA results indicated that the two-factor model fit the data better than the one-factor model. Logistic regression analyses indicated that being female, majoring in non-health-related disciplines, attributing homosexuality to uncontrollable causes, non-adherence to traditional gender norms and exposure to homosexual content were significantly associated with less negative attitudes toward both L and G. Urban students were marginally less likely to express negative attitudes toward L but not G, while non-heterosexuals and those who had prior personal contact with homosexuals exhibited less negative attitudes toward G but not L. However, grade showed no significant associations with either ATL or ATG. The retained 14-item version of Herek’s ATLG scale has been proven to be a reliable and valid tool. Furthermore, ATL and ATG were determined by different factors and thus would be treated separately. In order to reduce negative attitudes toward LG among undergraduates in mainland China, a comprehensive intervention plan such as conducting comprehensive sex education and pushing the process of legalizing same-sex marriage should be designed, implemented and evaluated.

## Introduction

Although lesbian women (L) and gay men (G) have no longer been diagnosed with a mental disorder because of their sexual identity, negative attitudes toward them continue to prevail in contexts such as families, hospitals, schools and workplaces (Fineran, 2002; Puckett et al., 2015; Ren et al., 2018; Corrêa-Ribeiro et al., 2019; Vecho et al., 2019; Pellegrini et al., 2020; Salvati et al., 2020). Negative attitudes can result in malicious comments, sexual harassment, and outright physical violence or discrimination against LG (Fineran, 2002; Puckett et al., 2015; Ren et al., 2018; Pellegrini et al., 2020). Furthermore, these negative experiences can be linked to internalized homophobia, contribute to both mental and physical health issues (Newcomb and Mustanski, 2010; Puckett et al., 2015; Elmer et al., 2018), and give rise to legal and moral concerns (Ren et al., 2018; Vecho et al., 2019). Therefore, eradication of prejudices against LG based on their sexual orientation is critical to develop harmonious interpersonal relationships, build safe communities and promote population health. Undergraduates have been frequently chosen as a key target population for anti-discrimination intervention, because they are open-minded to different opinions, viewpoints and values.

Several instruments have been attempted to measure undergraduates’ attitudes toward LG in mainland China (Yu et al., 2010, 2011; Song et al., 2013, 2014; Liu and Sun, 2015; Zhang et al., 2016; Chen et al., 2018; Liu, 2020). However, the lack of a standardized, reliable and valid method makes it difficult to measure consistently and thus poses a challenge to compare the effects of interventions. Thus, there is a need for developing a valid, reliable and globally accepted tool to assess the current level and determinants of negative attitudes toward LG, and subsequently develop, implement and evaluate educational programs to reduce sexual prejudices.

A review of the existing literature indicated that Herek’s ATLG scale, which was developed in 1988 and translated into Chinese language and also confirmed to have a two-dimensional structure [i.e., attitudes toward lesbian women (ATL) and attitudes toward gay men (ATG), as described in more detail below] in a sample of 2,391 participants by using confirmatory factor analysis (CFA) (Yu et al., 2011), seems to be a promising tool because it has the following three advantages. First, it distinguishes between ATL and ATG. Second, it has become one of the most widely used instruments for measuring negative attitudes of heterosexuals from various countries toward LG for more than three decades (Herek, 1988; Bas et al., 2003; Grigoropoulos et al., 2010; Yu et al., 2011; Delgado and Castro, 2012; Moreno et al., 2015; Corrêa-Ribeiro et al., 2019), due to its flexibility to adapt to different cultural, linguistic and historical contexts. Third, it has been well-validated in a relatively large sample of undergraduates (Yu et al., 2011), and applied to similar studies in mainland China (Song et al., 2014; Liu and Sun, 2015; Zhang et al., 2016; Liu, 2020).

While Herek’s ATLG scale was tested successfully in a sample of purely heterosexual undergraduates (i.e., measuring only sexual prejudice of heterosexuals) from Hunan province (Yu et al., 2011), its reliability and validity among general college students (including heterosexual and sexual minorities, i.e., measuring both sexual prejudice of heterosexuals and self-stigma of sexual minorities) throughout mainland China had not been fully tested prior to the current research. Furthermore, nine factors, including five demographic variables [i.e., gender (Yu et al., 2011; Liu and Sun, 2015; Zhang et al., 2016; Liu, 2020), sexual orientation (Liu and Sun, 2015), major (Chen et al., 2018), grade/age (Song et al., 2014; Liu, 2020), and residential areas (Song et al., 2014; Zhang et al., 2016)], prior contact with homosexuals (Song et al., 2014; Zhang et al., 2016; Liu, 2020), exposure to homosexual content (Song et al., 2014), adherence to traditional gender roles (Song et al., 2014) and causal attributions for homosexuality (Song et al., 2014; Zhang et al., 2016), have previously been studied as correlates of ATLG. However, most of these studies directly analyzed correlations between the independent variables and ATLG scores by using one-way ANOVA (or independent samples *t*-test) and multivariable linear regression. Few studies have tested the normality of ATLG scores and diagnosed the collinearity between the independent variables.

Therefore, the present study aimed to first validate Herek’s ATLG scale based on a large, nationally diverse sample of undergraduates and then apply rigorous statistical techniques to identify factors associated with negative attitudes toward LG. Based on the relevant literature, negative attitudes toward LG were hypothesized to be significantly associated with non-exposure to the content of homosexuality (Detenber et al., 2013), lack of contact with homosexuals, attributing homosexuality to controllable causes and adherence to traditional gender norms. It was also hypothesized that males and non-heterosexuals would be more likely to express negative attitudes toward LG. The role of the other three demographic variables (i.e., major, grade/age, and residential areas) was examined in a more exploratory fashion.

## Materials and methods

### Study design and setting

The cross-sectional survey was conducted between September 9, 2017 and December 31, 2017. The Ethics Committee at Hubei University of Science and Technology (HUST) approved the study and the Director of Students’ Affairs Division also provided a formal consent (No. 2021XG001) prior to conducting the survey. Participants were selected using a combination of convenience and snowball sampling techniques. Due to their convenience and better cooperation, undergraduates from HUST were first invited to complete the online questionnaire. Meanwhile, a series of measures such as earning extra credits and being rewarded the honor of outstanding volunteer were taken to encourage more undergraduates to participate in this survey. In addition, the research team also used social media (e.g., WeChat and Sina Weibo), relevant organizations and neighborhood groups to distribute the survey link in order to obtain a large national sample of college students.

Through the electronic consent form, participants were informed of the purpose of the study and of the fact that there were no right or wrong answers, were told that the survey was totally anonymous and no identifying information was included, and were also promised that they could withdraw from the survey at any time and all the information collected were only used for academic research. After signing the consent form, participants were asked to spend 10 min completing the questionnaire.

### Participants

In the online survey, a total of 6,954 respondents completed the questionnaires. However, to be eligible for the study, subjects had to be aged between 18 and 25 years old and enrolled as a full-time undergraduate student at one university in mainland China, and finished the questionnaire before the end of 2017.

### Measure

The structured questionnaire was developed from the conceptual, theoretical and empirical framework from previous studies by the Department of Preventive Medicine, and pilot tested with 50 students conveniently drawn from HUST.

### The dependent variable

The Chinese version (Yu et al., 2010) of Herek’s (1988) ATLG scale was used to measure attitudes toward LG. A detailed description of the wording of the items on this scale was shown in Table 1. As indicated in Table 1, the full ATLG scale consisted of 20 items, the first ten measuring attitudes toward lesbian women (ATL) and the next ten measuring attitudes toward gay men (ATG). Undergraduates responded to each item on a 5-point Likert scale ranging from 1 (strongly agree) to 5 (strongly disagree). Seven positively worded items (Items 2, 4, 7, 11, 15, 17, 20) require reverse scoring before being summed up to the total scores so that higher scores indicate more positive attitudes. Thus, ATL scores and ATG scores can range from 10 to 50 and their midpoint (30) equals a neutral attitude.

**TABLE 1:** Item wording, descriptive statistics and item-total correlations of the ATLG scale (*n* = 6036).

ATLG Item	*M*	*SD*	Skewness	Kurtosis	Shapiro-wilk test	ITC
1. Lesbians just can’t fit into our society.	3.51	0.98	–0.58	0.12	0.25***	0.71
2. A woman’s homosexuality should not be a cause for job discrimination in any situation. (R) (D)	3.73	0.98	–0.74	0.27	0.28***	**0.48**
3. Female homosexuality is detrimental to society because it breaks down the natural divisions between the sexes.	3.61	0.96	–0.62	0.16	0.26***	0.73
4. State Laws regulating private, consenting lesbian behavior should be loosened. (R) (D)	3.54	0.90	–0.47	0.16	0.25***	**0.59**
5. Female homosexuality is a sin.	3.74	0.88	–0.71	0.76	0.28***	0.78
6. The growing number of lesbians indicates a decline in American morals.	3.70	0.94	–0.72	0.40	0.28***	0.72
7. Female homosexuality in itself is no problem, but what society makes of it can be a problem. (R) (D)	3.85	0.88	–0.78	0.73	0.29***	**0.49**
8. Female homosexuality is a threat to many of our basic social institutions.	3.48	0.95	–0.45	–0.18	0.25***	0.70
9. Female homosexuality is an inferior form of sexuality.	3.71	0.91	–0.71	0.58	0.28***	0.78
10. Lesbians are sick.	3.69	0.94	–0.64	0.30	0.26***	0.78
11. Male homosexual couples should be allowed to adopt children the same as heterosexual couples. (R)	3.43	1.09	–0.34	–0.44	0.18***	0.63
12. I think male homosexuals are disgusting.	3.28	1.10	–0.33	–0.52	0.20***	0.77
13. Male homosexuals should not be allowed to teach school.	3.49	1.10	–0.59	–0.28	0.25***	0.69
14. Male homosexuality is a perversion.	3.68	1.02	–0.71	0.12	0.27***	0.80
15. Just as in other species, male homosexuality is a natural expression of sexuality in human men. (R)	3.44	0.99	–0.48	–0.21	0.25***	0.62
16. If a man has homosexual feelings, he should do everything he can to overcome them.	3.12	0.99	–0.21	–0.35	0.20***	0.68
17. I would not be too upset if I learned that my son were a homosexual. (R)	2.75	1.18	0.23	–0.97	0.23***	**0.56**
18. Homosexual behavior between two men is just plain wrong.	3.29	1.09	–0.45	–0.40	0.22***	0.75
19. The idea of male homosexual marriages seems ridiculous to me. (D)	3.22	1.03	–0.19	–0.08	0.23***	0.80
20. Male homosexuality is merely a different kind of lifestyle that should not be condemned. (R)	3.65	0.93	–0.68	0.44	0.27***	0.70

R, reverse-coded items; M, Mean; SD, Standard Deviation; ITC, Item-total correlations; ***P ≤ 0.001. The values highlighted in bold were lower than 0.6.

### Independent variables

As described in the background section, five demographic variables (i.e., gender, sexual orientation, residential areas, major and grade/age) were taken as independent variables. Consistent with Liu et al. (2019), participants were classified into heterosexuals, homosexuals, bisexuals, and those who were not sure of their sexual orientation identity based on their self-reported answer to the question “What is your sexual orientation?” In the final analysis, all participants in the last three groups were combined and fell under the category of non-heterosexual orientation due to their small sample size.

Undergraduates were asked whether they had known a homosexual person. Those who answered “yes” were classified as having prior contact with homosexuals, and those who answered “no” or “I don’t know” were classified as lack of contact (Badenes-Ribera et al., 2017; Salvati et al., 2019; Piumatti and Salvati, 2020). Additionally, the respondents were also classified into two groups based on their answers to the question about the origins of homosexuality: attributing it either to controllable factors (e.g., early life events, family environment and personal choices) or uncontrollable factors (i.e., genetic and biological causes) (Detenber et al., 2013; Costa et al., 2019; Vecho et al., 2019; Hermosa-Bosano et al., 2021).

In China from childhood, boys are taught to be strong, brave, ambitious and independent. On the other hand, girls are usually taught to be gentle and compliant. Homosexuality is often perceived as having cross-gender traits, roles, and physical characteristics (Whitley and Ægisdóttir, 2000) and potentially threaten family values and traditional lifestyles (Ren et al., 2018). In order to measure their attitudes toward traditional gender norms, respondents were asked to indicate what characteristics women and men should exhibit and also provided with four choices: ➀Men should exhibit masculine personality traits and women should display feminine personality traits; ➁Men can display feminine personality traits; ➂Women can exhibit masculine personality traits; ➃Everyone shows a mix of masculine and feminine personality traits, irrespective of their gender. In the final analysis, only those who held the view that masculine personality traits are stereotypically associated with men and feminine personality traits were stereotypically associated with women were categorized as adherence to traditional gender norms, while their counterparts were categorized as non-adherence to traditional gender norms.

Gay men can be classified into three groups: insertive (“1” = Top), receptive (“0”= Bottom), or both (“0.5” = Versatile), based on their sexual behaviors (Moskowitz et al., 2021). Similarly, there are three types of sex role preferences (i.e., T, P, and H) in the lesbian community. T represents an abbreviation for “Tomboy,” who identifies as a male, while “Pourgirl” is commonly abbreviated as P and comes from a Taiwanese slang “pó” (婆, meaning “a female” in Chinese) (Chen and Chen, 2007). H is an abbreviation for “Half” which refers to those who do not designate as either T or P, or those who can play either of the roles according to their partner’s role. In this study, respondents were asked whether they had knowledge about the terms of sexual preferences (i.e., 1, 0, 0.5, T, P, and H). Undergraduates were defined as non-exposure to homosexual content if they knew nothing about these terms, and were defined as exposure to homosexual content otherwise.

### Statistical analysis

The data analysis was conducted in stages. First, a detailed analysis of the sample was undertaken and item analysis was used to remove poorly performing or statistically redundant items. The sample was then randomly split in half and exploratory factor analysis (EFA) was conducted on the first data set, followed by CFA and reliability analysis on the second data set. Logistic regression analyses were finally carried out to identify factors affecting negative attitudes toward LG. Except for the CFA, which was performed using Amos 25, all other data analyses were performed by SPSS version 25.0.

## Results

### Characteristics of participants

**FIGURE 1:**
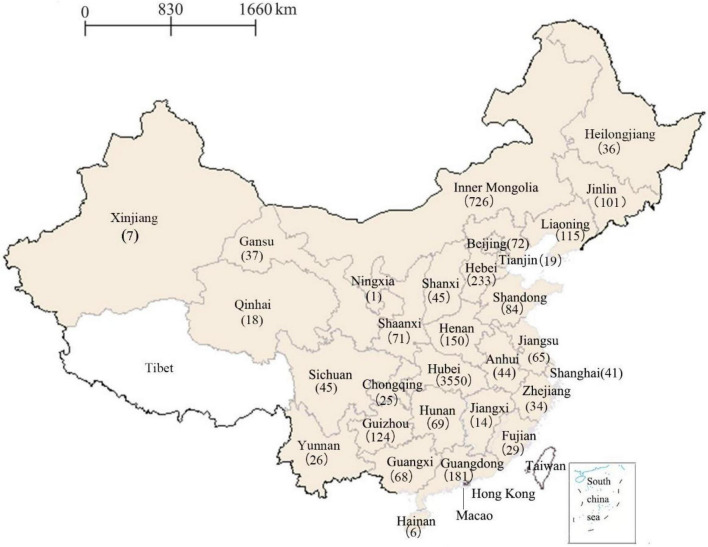
A map displaying the provincial distribution of 6,036 undergraduates was drawn using Supermap iDesktop 8C (2017) and then converted into Microsoft Word format. Excluding Taiwan, Hong Kong, and Macao, there are 31 provinces in mainland China. The exact number in the map indicated that the 6,036 participants were unevenly distributed across 30 provinces (except for Tibet with white highlighted), and were mainly (3,550) recruited from Hubei province.

A total of 6,954 completed questionnaires were received and the effective data utilization rate was 86.8% (6,036/6,954) after excluding 918 invalid questionnaires. As indicated in Figure 1, the eligible 6,036 students were unevenly distributed across China’s 30 provinces, municipalities and autonomous regions (excluding Tibet, Hongkong, Macao, and Taiwan) and the majority (58.8%, 3,550/6,036) of enrolled participants were from Hubei province. Table 2 shows social-demographic characteristics and homosexuality-related values, beliefs and behaviors of the 6,036 undergraduates in mainland China. As shown in Table 2, more than three-fifths (63.4%) of participants were females, 50.5% were from rural areas, 12.6% were self-identified as non- heterosexuals, and 40.1% majored in health-related disciplines such as nursing, medicine and psychology. More than one-third (37.1%) had already completed more than 2 years of college study (i.e., juniors and seniors). Beyond our expectation, nearly four-fifths (77.0%) of students attributed homosexual orientation to controllable factors such as early life events, family environment and personal choices, 55.5% adhered to traditional gender norms, 57.4% had no prior contact with homosexuals, and 60.7% were not exposed to LG content (i.e., sex-role-preferences).

**TABLE 2:** Social-demographic characteristics and homosexuality-related values, beliefs and behaviors of the 6,036 undergraduates in mainland China.

Variable	Total (*n* = 6036)		Sample1 (*n* = 3001)		Sample 2 (*n* = 3,035)		χ^2^	*P*
	*n*	%	*n*	%	*n*	%		
**X1: Gender**								
0 = Male	2,207	36.6	1,082	36.1	1,125	37.1	0.67	0.414
1 = Female	3,829	63.4	1,919	63.9	1,910	62.9		
**X2: Sexual orientation**								
0 = Heterosexuals	5,275	87.4	2,619	87.3	2,656	87.5	0.08	0.778
1 = Non-Heterosexuals	761	12.6	382	12.7	379	12.5		
**X3: Residential areas**								
0 = Rural	3,050	50.5	1,524	50.8	1,526	50.3	0.15	0.696
1 = Urban	2,986	49.5	1,477	49.2	1,509	49.7		
**X4: Major**								
0 = Health-related^&^	2,422	40.1	1,221	40.7	1,201	39.6	0.78	0.377
1 = Others	3,614	59.9	1,780	59.3	1,834	60.4		
X5: Grade								
0 = Low	3,797	62.9	1,909	62.2	1,888	63.6	1.28	0.259
1 = High	2,239	37.1	1,092	37.8	1,147	36.4		
**X6: Attributions**								
0 = Controllable	4,647	77.0	2,298	76.6	2,349	77.4	0.58	0.448
1 = Uncontrollable	1,389	23.0	703	23.4	686	22.6		
**X7: Traditional gender norms**								
0 = Adherence	3,352	55.5	1654	55.1	1,698	55.9	0.42	0.515
1 = Non-adherence	2,684	44.5	1347	44.9	1,337	44.1		
X8: Personal contact								
0 = Lacking	3,465	57.4	1,708	56.9	1,757	57.9	0.59	0.443
1 = Having	2,571	42.6	1,293	43.1	1,278	42.1		
**X9: Exposure to homosexual content**								
0 = Non-exposed	3,661	60.7	1,838	61.2	1,823	60.1	0.88	0.348
1 = Exposed	2,375	39.3	1,163	38.8	1,212	39.9		

^&^Including nursing, preventive medicine, clinical medicine, social medicine and health psychology.

The final sample size of the derivation sample (sample 1) was 3,001 and the confirmatory sample (sample 2) was 3,035. Table 2 also indicated that there were no statistical difference between sample 1 and sample 2 across all social-demographic characteristics and homosexuality-related values, beliefs and behaviors.

### Item analysis

From Table 1, it was observed that correlation coefficients between single scores of the second (*r* = 0.48), fourth (*r* = 0.59), seventh (*r* = 0.49), and seventeenth (*r* = 0.56) items and the overall score were less than 0.60. Meanwhile, the inter-item correlation matrix (Table 3) indicated that a strong correlation (i.e., inter-item correlations were larger than 0.70) existed between items 5 and 9 (*r* = 0.71), 9 and 10 (*r* = 0.79), and 18 and 19 (*r* = 0.72). Therefore, six items (Item 2, 4, 7, 9, 17 and 19) were removed from the original scale due to low item-total correlations [*r* < 0.60, (Chen et al., 2018)] or redundancy [(*r* > 0.70, Sousa et al., 2010)] and the following statistical analyses were carried out on the retained 14- item version of Herek’s ATLG scale.

**TABLE 3:** Inter-item correlation matrix of the ATLG scale.

	C1	C2	C3	C4	C5	C6	C7	C8	C9	C10	C11	C12	C13	C14	C15	C16	C17	C18	C19	C20
C1	–																			
C2	0.28	–																		
C3	0.59	0.30	–																	
C4	0.37	0.42	0.37	–																
C5	0.61	0.34	0.67	0.41	–															
C6	0.54	0.29	0.61	0.35	0.70	–														
C7	0.29	0.41	0.32	0.45	0.36	0.33	–													
C8	0.50	0.25	0.60	0.33	0.61	0.62	0.26	–												
C9	0.58	0.34	0.63	0.41	**0.71**	0.67	0.34	0.64	–											
C10	0.60	0.33	0.61	0.42	0.68	0.64	0.36	0.61	**0.79**	–										
C11	0.34	0.27	0.34	0.38	0.35	0.32	0.26	0.33	0.37	0.39	–									
C12	0.51	0.24	0.51	0.34	0.53	0.47	0.25	0.48	0.53	0.56	0.50	–								
C13	0.47	0.26	0.46	0.31	0.49	0.46	0.23	0.44	0.50	0.50	0.41	0.59	–							
C14	0.54	0.29	0.55	0.35	0.60	0.55	0.31	0.52	0.60	0.61	0.45	0.69	0.63	–						
C15	0.33	0.30	0.35	0.41	0.38	0.33	0.33	0.32	0.37	0.37	0.48	0.44	0.35	0.45	–					
C16	0.46	0.20	0.47	0.30	0.48	0.44	0.20	0.44	0.47	0.46	0.39	0.56	0.46	0.56	0.36	–				
C17	0.29	0.23	0.29	0.34	0.29	0.26	0.23	0.29	0.30	0.33	0.45	0.44	0.33	0.37	0.44	0.39	–			
C18	0.51	0.22	0.51	0.37	0.53	0.48	0.25	0.47	0.53	0.53	0.44	0.63	0.51	0.62	0.45	0.59	0.42	–		
C19	0.55	0.26	0.55	0.38	0.57	0.52	0.26	0.51	0.56	0.58	0.50	0.68	0.56	0.66	0.44	0.61	0.46	**0.72**	–	
C20	0.43	0.43	0.42	0.51	0.46	0.40	0.43	0.38	0.46	0.45	0.49	0.48	0.42	0.53	0.51	0.41	0.43	0.48	0.51	–

The values highlighted in bold were higher than 0.7.

### Exploratory factor analysis

The Sample 1 (*n* = 3001) was considered to be appropriate for factor analysis, because the KMO value was 0.96 (close to 1) and Bartlett’s Test of Sphericity was also highly significant (χ^2^ = 23744.39, *df* = 91, and *p*< 0.001). Two factors emerged with an eigenvalue greater than one, which accounted for 61.12% of the total variance in the data. As indicated in Table 4, all items were successfully assigned to the given factor as expected from the original model (i.e., six items measured ATL and eight items measured ATG). In addition, all items had an acceptable factor loading (≥0.50) on a single factor. The factor loading of each item, detailed eigenvalue and explained variance of each loaded factor were shown in Table 4.

**TABLE 4:** Results of the EFA and internal consistency of the ATLG scale.

Item code	Factor Loading	
	ATL	ATG
C1	0.59	
C3	0.70	
C5	0.76	
C6	0.75	
C8	0.67	
C10	0.70	
C11		0.60
C12		0.73
C13		0.59
C14		0.69
C15		0.55
C16		0.57
C18		0.65
C20		0.56

Eigenvalue	7.35	1.21
% of variance explained by before rotation	52.48	8.63
Cronbach’s alpha (Total = 0.93)	0.90	0.88

### Confirmatory factor analyses

The CFA was conducted on the second half of the sample (*n* = 3035) to confirm the two-factor structure identified *via* the above EFA against a one-factor model. As reported in Table 5, although the χ^2^-test was statistically significant (χ^2^ = 1021.84, *df* = 76, *p* < 0.001), other indexes indicated that the two-factor model had a reasonable fit to the data: *GFI* = 0.95, *CFI* = 0.96, *NFI* = 0.96, and R*MSEA* = 0.06. Furthermore, the goodness-of-fit indices of the two models showed that the two-factor model fit better than the one-factor model. Consequently, the two-factor model was finally accepted.

**TABLE 5:** Fitting indices of model (*n* = 3035).

Model	χ^2^	df	χ^2^/df	GFI	NFI	CFI	RMSEA
One factor	3169.66	90	35.22	0.83	0.88	0.89	0.11
Two-factor	1021.84	76	13.44	0.95	0.96	0.96	0.06

**FIGURE 2:**
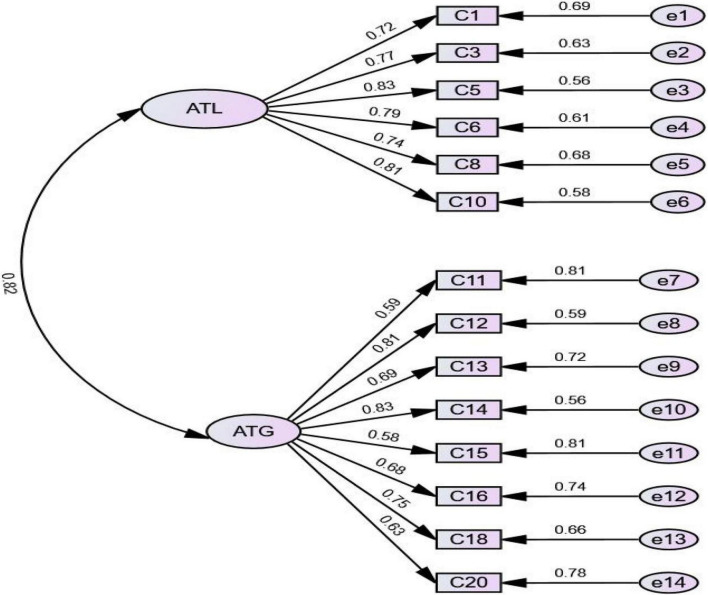
Confirmatory factor analysis: standardized estimates (*n* = 3035).

As shown in Figure 2, all of the items significantly loaded onto the same factor in the CFA as they had in the EFA and all of the standardized factor loadings of the two- factor model were also above 0.50. Two derived subscales (i.e., ATL and ATG) were distinct, yet related (*r* = 0.82, *p* < 0.001), and thus separate analyses were required.

### Reliability

The Cronbach’s alpha value of the revised 14-item scale was estimated to be 0.93 for ATLG, 0.90 for ATL; and 0.88 for ATG. The ATLG and the two subscales demonstrated good internal consistence for this sample.

### Factors associated with negative attitudes toward LG people

ATL scores and ATG scores were not normally distributed (according to the Shapiro-Wilk test; all *Ps* < 0.001) (see Table 1) and therefore categorized into three groups based on their midpoints. In this survey, 13.4% of undergraduates expressed negative attitudes toward L, 14.2% were neutral, and 72.4% expressed positive attitudes. In contrast, 22.5% exhibited negative attitudes toward G, 10.1% were neutral, and 67.4% exhibited positive attitudes. Therefore, undergraduates expressed significantly more negative attitude (χ^2^ = 173.24, *p*< 0.001) toward G than toward L.

### Multicollinearity diagnosis

The VIF values of each independent variable ranging between 1.01 and 1.19 were much smaller than 10, indicating the absence of multicollinearity (Table 6). Therefore, all the nine variables were entered into multivariate Logistic regressions to control the effects of confounding factors.

**TABLE 6:** Logistic regression analyses of factors associated with ATL and ATG (*n* = 6,036).

Independent Variables	VIF	ATL		ATG	
		OR	95% CI	OR	95% CI
Gender (0 = Male, 1 = Female)	1.04	0.40***	0.34–0.47	0.33***	0.29–0.37
Sexual orientation (Ref:Heterosexual)	1.12	0.89	0.68–1.18	0.63***	0.48–0.82
Residential area (0 = Rural, 1 = Urban)	1.06	0.85*	0.72–0.99	0.88	0.77–1.01
Major (Ref: Health-related)	1.01	0.75***	0.64–0.87	0.77***	0.67–0.88
Grade (0 = Low, 1 = High)	1.01	1.06	0.90–1.24	1.06	0.92–1.21
Attribution (Ref: Controllable)	1.04	0.71***	0.58–0.87	0.62***	0.52–0.74
Norms (Ref: Adherence)	1.10	0.52***	0.44–0.62	0.39***	0.34–0.45
Contact (0 = Lacking, 1 = Having)	1.12	1.13	0.96–1.34	0.81**	0.70–0.93
Exposure (0 = Non-exposed, 1 = Exposed)	1.19	0.75***	0.63–0.90	0.53***	0.46–0.62

*P ≤ 0.05, **P ≤ 0.01, and ***P ≤ 0.001.

### Factors associated with negative attitudes toward LG

Separate Logistic regression analyses were performed to identify significant variables affecting negative attitudes toward LG. As indicated in Table 6, five factors were found to be significantly associated with ATL and ATG. More specifically, being female (*AOR* = 0.40, *95% CI*: 0.34–0.47; *AO*R = 0.33, *95% CI:* 0.29–0.37, respectively), majoring in non-health-related disciplines (*AOR* = 0.75, *95% CI*: 0.64–0.87; *AOR* = 0.77, 95% *CI*: 0.67–0.88, respectively), attributing homosexuality to uncontrollable causes (*AOR* = 0.71, *95% CI*: 0.58–0.87; *AOR* = 0.62, *95% CI*: 0.52–0.74, respectively), non- adherence to traditional gender norms (*AOR* = 0.52, *95% CI*: 0.44–0.62; *AOR* = 0.39, *95% CI*: 0.34–0.45, respectively) and exposure to homosexual content (*AOR* = 0.75, *95% CI*: 0.63–0.90; *AOR* = 0.53, *95% CI*:0.46–0.62, respectively) were significantly associated with less negative attitudes toward both L and G. Urban students (*AOR* = 0.85, *95% CI*: 0.72–0.99, *p* = 0.035) were marginally less likely than rural students to express negative attitudes toward L but not G, while non-heterosexuals (AOR = 0.63, 95% CI: 0.48–0.82) and those who had prior contact with homosexuals (*AOR* = 0.81, *95% CI*: 0.70–0.93) exhibited less negative attitudes toward G but not L. However, grade showed no significant associations with either ATL or ATG.

## Discussion

The first aim of the present study was to test the validity and reliability of Herek’s ATLG scale among a large, nationally diverse sample of undergraduates. Based on the item analysis of the 20 items, six items (Item 2, 4, 7, 9, 17, and 19) were removed from the original scale resulting in a short 14-item scale. EFA supported the existence of two factors (ATL and ATG), as was theoretically expected (Herek, 1988; Yu et al., 2011; Kenig, 2019): six items measuring ATL and eight items related to ATG. CFA results indicated that the two-factor model fit the data better than the one-factor model. The short version of the scale also demonstrated adequate reliability in internal consistency. Our findings were supported by previous studies which indicated that some items were not clearly expressed or not appropriate in some cultural contexts (Bas et al., 2003; Moreno et al., 2015; Corrêa-Ribeiro et al., 2019). More specifically, Item 4 (“State laws regulating private, consenting lesbian behavior should be loosened”) was not applicable in Chinese context, because just like Brazil and the Netherlands, China has no state law regulating homosexual behavior (Bas et al., 2003; Corrêa-Ribeiro et al., 2019). Similarly, given the increasing tolerance of homosexuality (Altemeyer, 2002; Detenber et al., 2013), it was recommended by Bas et al. (2003) and Corrêa-Ribeiro et al. (2019) to modify or eliminate “extreme words” such as “too” in item 17 (“I would not be too upset if I learned that my son were a homosexual”) and “in any situation” in item 2 (“A woman’s homosexuality should not be a cause for job discrimination in any situation”). Furthermore, Moreno et al. (2015) found that the omission of item 4 (“Female homosexuality in itself is no problem, but what society makes of it can be a problem”) yielded in increase in Cronbach’s α. More importantly, Herek and McLemore (2011) recommended to use the shorter versions instead of the original version, because these short versions were highly correlated with the original version, and in recent years the shorter versions (e.g., the five- item versions of the ATL and ATG scale) have been used more and more frequently (Herek and Gonzalez-Rivera, 2006; Song et al., 2013; Zhang et al., 2016; Kenig, 2019).

In terms of factors associated with ATLG, our results were largely consistent with findings from previous studies (Herek and Gonzalez-Rivera, 2006; Herek and McLemore, 2011; Yu et al., 2011; Zhang et al., 2016; Hermosa-Bosano et al., 2021). More specifically, gender, major, attribution of controllability, adherence to traditional gender norms and exposure to homosexual content were significantly associated with both ATL and ATG. Residential area was significantly associated with ATL but not with ATG, while sexual orientation and prior contact with homosexuals were significantly associated with ATG but not with ATL. However, grade showed no significant association with either ATL or ATG. These findings further indicate that ATL and ATG are similar, but there are some subtle differences between them.

Consistent with previous studies (Herek and Gonzalez-Rivera, 2006; Yu et al., 2011; Liu and Sun, 2015; Zhang et al., 2016; Liu, 2020; Hermosa-Bosano et al., 2021), men were more likely than women to express negative attitudes toward LG (in particular homosexuals of the same gender, i.e., gay men). This finding can be explained by the fact that machismo culture still prevails in mainland China. Amid the machismo culture, men are more likely to face strong pressures to affirm their own heterosexuality and masculinity by constructing hostile attitudes toward G.

Self-stigma is defined by Herek et al. (2015) as sexual minorities’ negative attitude toward their own status as a member of a stigmatized group, while sexual prejudice is defined as prejudice against sexual minorities by heterosexuals on the basis of sexual orientation. Lesbians (L) and gay men (G), who are commonly considered to violate traditional gender roles, were also found to face discrimination and prejudice in gay and lesbian communities (Salvati et al., 2018, 2021). Consistent with previous studies (Liu and Sun, 2015; Hermosa-Bosano et al., 2021), heterosexuals were more likely to exhibit negative attitudes toward G. However, no statistically significant difference existed between these two groups in attitudes toward L.

The effect of residential area was inconsistent with a previous study (Song et al., 2014) in which undergraduates from urban areas were more tolerant of G than those from rural areas. This could be partially explained by the fact social and cultural norms were deeply rooted in some people’s mind, especially in distant rural areas. However, in recent years, China has experienced rapid urbanization and industrialization growth, thus contributing to the narrowing of urban-rural differences. Consequently, our finding indicated urban students were less likely than their rural counterparts to exhibit negative attitudes toward LG, but there was no statistically significant difference between these two groups in attitudes toward G.

Education enables individuals to be exposed to different cultures and contributes to more tolerance and open-mindedness. Previous studies have suggested that individuals with greater levels of education expressed less negative attitudes toward LG (Herek and Gonzalez-Rivera, 2006; Yu et al., 2011). Beyond our expectation, undergraduates from health-related disciplines were found to be less tolerant of LG than their counterparts, possible due to the fact that medical course usually associates AIDS with homosexuality (Zheng et al., 2016; Ruan et al., 2019), and that this association might be related to higher levels of anti-homosexual attitude (Herek and McLemore, 2011). Consistent with previous studies (Herek and Gonzalez-Rivera, 2006; Fetner, 2008; Detenber et al., 2013; Song et al., 2014), older students (i.e., seniors and juniors) expressed more negative attitudes toward LG than younger students (i.e., freshmen and sophomore). However, the subtle difference in their ages may result in statistically insignificant difference between these two groups.

Consistent with previous studies (Sakalli, 2002; Detenber et al., 2013; Song et al., 2014; Zhang et al., 2016; Badenes-Ribera et al., 2017; Hermosa-Bosano et al., 2021), undergraduates who believed that the root cause of homosexuality is genetic (i.e., uncontrollable) expressed less negative attitudes toward LG, compared with those who believed that homosexuality is a learned characteristic (controllable). This finding is not surprising since it conforms to Weiner’s attribution theory of controllability, which enables us to systematically examine how individuals or groups attribute homosexuality. However, it should be noted that the percentage of college students who held the belief that homosexuality is uncontrollable is still very low. Therefore, the scientific evidence on biological origins of sexual orientation should be made accessible to college students to undermine moral attacks on homosexuality.

In the Chinese cultural contexts, heterosexuality is the norm or preferred sexual orientation, while homosexuality is often seen to deviate from social norms and potentially threaten family values and traditional lifestyles (Ren et al., 2018). Our analysis is therefore consistent with previous studies (Sakalli, 2002; Detenber et al., 2013; Song et al., 2014) that indicate respondents who adhered to traditional gender norms were more intolerant of homosexuals.

In line with previous studies (Detenber et al., 2013; Song et al., 2014; Liu, 2020; Hermosa-Bosano et al., 2021), our findings have suggested that exposure to homosexual content or having prior contact with homosexuals might contribute to greater tolerance of homosexuals. Given the fact that less than half of them were exposed to homosexual content or had a homosexual friend, undergraduates should be recommended to read materials depicting homosexuality in a positive way (e.g., explaining that homosexuality is the same as heterosexuality and is also a normal sexual orientation) or increase positive encounters with LG (Altemeyer, 2002; Salvati et al., 2019) such as inviting some volunteers to share their personal stories (Eick et al., 2016) to dispel myths, reduce fear, promote mutual understanding and finally reduce prejudice toward homosexual individuals.

### Limitations and future directions

Several limitations of the present study need to be taken into account. First, the cross-sectional nature of this study limits the ability to claim causality. In order to verify their causal relationships, case-control studies, cohort studies, even randomized controlled trials and systematic reviews should be conducted to obtain more reliable evidences. Second, this study used a convenience sample and might affect the generalizability of its findings. Third, no identifying information was collected to preserve individual confidentiality, thus making it impossible to assess the test-retest reliability. Furthermore, criterion validity could not be assessed, because there is no gold standard to measure attitudes toward LG. Fourth, sexual minorities are a heterogeneous group composed of lesbians, gay men, bisexual men, bisexual women, male-to-female (MtF) and female- to-male (FtM) transgender (LGBT) individuals. Previous studies also indicated that individual respondents reacted very differently to lesbians, gay men, bisexual women and bisexual men (Vaughn et al., 2016). It is therefore recommended that, when examining attitudes toward sexual minorities, we should investigate attitudes toward each group individually, rather than just exploring attitudes toward sexual minorities as a whole (e.g., using the commonly used term “homosexual”), or differentiating male or female homosexuals in this study. Fifth, just a single item was used to assess prior contact with homosexuals. According to Black and Stevenson (1984), respondents often equated homosexuality with male homosexuality (i.e., gay men). Furthermore, it does not assess the characteristics of the relationship (e.g., acquaintance, friends, colleagues or family members), number of LG known and the frequency of contact (Vecho et al., 2019; Hermosa-Bosano et al., 2021). The sixth limitation of this study is that the data were collected before the end of 2017. China might have undergone many sociocultural and legal changes over the past 5 years. For example, Liu Hua, a special representative for Human Rights of Ministry of Foreign Affairs of China, on October 24, 2019 said that China opposes all forms of discrimination and violence, including discrimination, violence and intolerance based on sexual orientation. These changes might have led to a more favorable attitude toward LG and have also resulted in increases or decreases in some variables (Detenber et al., 2013). However, a recent survey conducted in China has shown that discrimination based on sexual orientation still existed on college campuses (Wang et al., 2020). And many aspects in this field (e.g., lack of a standardized reliable and valid instrument to measure negative attitudes toward LG) need to be improved and strengthened (Yang, 2020). Therefore, the results of this study added to the literature on the validation of Herek’s ATLG scale. Finally, other potential influences which have not been studied extensively in this study included political orientation and religious involvement (Delgado and Castro, 2012; Vecho et al., 2019) as well as attachment styles, openness to experience and other social and psychological variables (Metin-Orta and Metin-Camgöz, 2020). Therefore, further studies should be conducted to quantify the relative contributions of political, socioeconomic, cultural, and biological factors to negative attitudes toward LG.

### Implications of the study

Our findings have several important implications. First, conduct comprehensive sex education (Leos and Wiley, 2019). Early in 2017, the guideline issued by the Ministry of Education called on higher education institutions to set up courses to teach their students about sexual and reproductive health knowledge. Also, the newly revised Law on the Protection of Minors, which took effect from June 1, 2021, stipulates that schools should conduct comprehensive sex education. However, China, as a traditionally conservative country (Ren et al., 2018), still faces serious challenges in providing comprehensive sex education. In order to achieve the goal of gender equality, college students (peers), teachers, parents, social workers and healthcare professionals should be trained to communicate more effectively with adolescents about reasons for sexual diversity and attitudes toward individuals with non- heterosexual orientations (Macintyre et al., 2015; Heras-Sevilla et al., 2021).

Second, push the process of legalizing same-sex marriage. As a result of globalization, the idea of recognizing same-sex marriage is expanding rapidly throughout the world. Until now, some countries such as Canada, the Netherlands, Belgium and Spain legalized same-sex marriages nationwide. Furthermore, Taiwan has become the first place in Asia to legalize same-sex unions. However, there has been no nationwide laws allowing same-sex marriage in China. Fortunately, Li Yinhe, a renowned social scientist, repeatedly submitted proposals suggesting the legalization of same-sex marriage. And it is firmly believed that China will consider to grant legal recognition to same-sex marriages to follow the worldwide trend.

## Conclusion

To the best of our knowledge, ours is the first to validate Herek’s ATLG scale among a large, nationally diverse sample of undergraduates and to examine factors associated with negative attitudes toward LG in this population. Our results indicated that the retained 14-item version of the scale is composed of two distinct subscales for separate assessment of attitudes toward lesbians and gay men, and can serve as a reliable and valid measurement tool for identifying undergraduates with high levels of sexual prejudice. Furthermore, ATL and ATG were determined by different factors and thus would be treated separately. In order to reduce negative attitudes toward LG among undergraduates in mainland China, a comprehensive intervention plan such as conducting comprehensive sex education and pushing the process of legalizing same- sex marriage should be designed, implemented and evaluated.

## Data availability statement

The datasets presented in this study can be found in online repositories. The names of the repository/repositories and accession number(s) can be found below: https://dataverse.harvard.edu/dataset.xhtml?persistentId=doi:10.7910/DVN/MGHNGE.

## Ethics statement

The studies involving human participants were reviewed and approved by The Ethics Committee at Hubei University of Science and Technology. The patients/participants provided their written informed consent to participate in this study.

## Author contributions

All authors have made substantial contributions to the conception of the work, as well as the analysis and interpretation of its data, reviewed the work, given their final approval of the version to be published, agreed to be accountable for the content of the work, contributed to the manuscript and agreed with the submission.

## References

[B1] Altemeyer B. (2002). Changes in attitudes toward homosexuals. *J. Homosex.* 42 63–75. 10.1300/J082v42n02_04 12013575

[B2] Badenes-Ribera L. Frias-Navarro D. Berrios-Riquelme J. Longobardi C. (2017). Italian validation of the queer/liberationist scale (short version) in a sample of university students: Confirmatory factor analysis. *Sex. Res. Soc. Policy* 14 157–170. 10.1007/s13178-016-0256-7

[B3] Bas V. D. M. Eisinga R. Felling A. (2003). Application of Herek’s attitudes toward lesbians and gay men scale in the Netherlands. *Psychol. Rep.* 93 265–275. 10.2466/pr0.2003.93.1.265 14563061

[B4] Black K. N. Stevenson M. R. (1984). The relationship of self-reported sex-role characteristics and attitudes toward homosexuality. *J. Homosex.* 10 83–93. 10.1300/J082v10n01_06 6520390

[B5] Chen C. Zheng G. Zhou H. Jiang H. (2018). Formed psychometrics measuring college students’ repellency to gays/lesbians: Based on social distance. *Sichuan Univ. Arts Sci. J.* 28 120–125.

[B6] Chen Y. Chen Y. (2007). Lesbians in China’s mainland. *J. Lesbian Stud.* 10 113–125. 10.1300/j155v10n03_08 17210562

[B7] Corrêa-Ribeiro R. Iglesias F. Camargos E. F. (2019). Attitudes toward lesbians and gay men scale: Validation in Brazilian physicians. *Einstein* 17:eAO4527. 10.31744/einstein_journal/2019AO4527 PMC6497123 31066793

[B8] Costa P. A. Pereira H. Leal I. (2019). Through the lens of sexual stigma: Attitudes toward lesbian and gay parenting. *J. GLBT Fam. Stud.* 15 58–75. 10.1080/1550428X.2017.1413474

[B9] Delgado J. B. Castro M. C. (2012). A confirmatory factor analysis of the Spanish language version of the attitudes toward lesbians and gay men scale (ATLG). *Univ. Psychol.* 11 579–586. 10.1111/j.1360-0443.2005.01124.x 16042648

[B10] Detenber B. H. Ho S. S. Neo R. L. Malik S. Cenite M. (2013). Influence of value predispositions, interpersonal contact, and mediated exposure on public attitudes toward homosexuals in Singapore. *Asian J. Soc. Psychol.* 16 181–196. 10.1111/ajsp.12006

[B11] Eick U. Rubinstein T. Hertz S. Slater A. (2016). Changing attitudes of high school students in Israel toward homosexuality. *J. LGBT Youth* 13 192–206. 10.1080/19361653.2015.1087930

[B12] Elmer E. Van T. T. Fokkema T. (2018). Chronic loneliness among lesbian, gay, and bisexual older adults: Impact of early-life and current marginalization. *Innov. Aging* 2(Suppl. 1):909. 10.1093/geroni/igy031.3384

[B13] Fetner A. T. (2008). Cohort differences in tolerance of homosexuality. *Public Opin. Q.* 72 311–330. 10.1093/poq/nfn017

[B14] Fineran S. (2002). Sexual harassment between same-sex peers: Intersection of mental health, homophobia, and sexual violence in schools. *Soc. Work* 47 65–74. 10.1093/sw/47.1.65 11829246

[B15] Grigoropoulos I. Papaharitou S. Moraitou M. (2010). Adaptation of the attitudes toward lesbians and gay men (ATLG) scale into the Greek language. *Arch. Hell. Med.* 27 787–792.

[B16] Heras-Sevilla D. Ortega-Sánchez D. Rubia-Avi M. (2021). Coeducation and citizenship: A study on initial teacher training in sexual equality and diversity. *Sustainability* 13:5233. 10.3390/su13095233

[B17] Herek G. M. (1988). Heterosexuals’ attitudes toward lesbians and gay men: Correlates and gender differences. *J. Sex. Res.* 25 451–477. 10.1080/00224498809551476

[B18] Herek G. M. Gillis J. R. Cogan J. C. (2015). Internalized stigma among sexual minority adults: Insights from a social psychological perspective. *Stigma Health* 1 18–34.

[B19] Herek G. M. Gonzalez-Rivera H. M. (2006). Attitudes toward homosexuality among U.S. residents of Mexican descent. *J. Sex. Res.* 43 122–135. 10.1080/00224490609552307 16817059

[B20] Herek G. M. McLemore K. A. (2011). “Attitudes toward lesbians and gay men scale,” in *Handbook of sexuality-related measures*, 3rd Edn, eds Fisher T. D. Davis C. M. Yarber W. L. Davis S. L. (New York, NY: Routledge), 415–417.

[B21] Hermosa-Bosano C. Hidalgo-Andrade P. Olaya-Torres A. Duque-Romero C. Costa P. A. Salinas-Quiroz F. (2021). Attitudes toward lesbians, gay men, and their rights in a sample of Ecuadorian cisgender men and women. *J. Homosex.* 10.1080/00918369.2021.1948771 34283008

[B22] Kenig N. (2019). *Psychometric analysis of the short version of attitudes toward lesbians and gay men scale (ATLG-S)*. Cyril: Methodius University in Skopje. 10.37510/godzbo1972169k

[B23] Leos C. Wiley D. (2019). “It falls on all our shoulders”: Overcoming barriers to delivering sex education in West Texas schools. *J. Appl. Res. Child.* 10:4.

[B24] Liu J. Sun L. (2015). Research on the cognitive attitude of university students to homosexuality. *Chin. J. Health Psychol.* 23 1700–1704.

[B25] Liu X. (2020). An investigation of attitudes toward gay men and lesbians among college students. *J. Campus Life Ment. Health* 18 121–122.

[B26] Liu Y. Yang M. Zhao C. Tan S. Tang K. (2019). Self-identified sexual orientations and high-risk sexual behaviours among Chinese youth. *BMJ Sex. Reprod. Health* 45 255–262. 10.1136/bmjsrh-2018-200150 31413158

[B27] Macintyre A. Vega A. Sagbakken M. (2015). “Sexuality? A million things come to mind”: Reflections on gender and sexuality by Chilean adolescents. *Reprod. Health Matter.* 23 85–95. 10.1016/j.rhm.2015.11.003 26719000

[B28] Metin-Orta I. Metin-Camgöz S. (2020). Attachment style, openness to experience, and social contact as predictors of attitudes toward homosexuality. *J. Homosex.* 67 528–553. 10.1080/00918369.2018.1547562 30507289

[B29] Moreno A. Herazo E. Oviedo H. Campo-Arias A. (2015). Measuring homonegativity: Psychometric analysis of Herek’s attitudes toward lesbians and gay men scale (ATLG) in Colombia, South America. *J. Homosex.* 62 924–935. 10.1080/00918369.2014.1003014 25569818

[B30] Moskowitz D. A. Avila A. A. Kraus A. Birnholtz J. Macapagal K. (2021). Top, bottom, and versatile orientations among adolescent sexual minority men. *J. Sex. Res.* 59 643–651. 10.1080/00224499.2021.1954583 34309441

[B31] Newcomb M. E. Mustanski B. (2010). Internalized homophobia and internalizing mental health problems: A meta-analytic review. *Clin. Psychol. Rev.* 30 1019–1029. 10.1016/j.cpr.2010.07.003 20708315

[B32] Pellegrini V. De Cristofaro V. Giacomantonio M. Salvati M. (2020). Why are gay leaders perceived as ineffective? The role of the type of organization, sexual prejudice and gender stereotypes. *Pers. Individ. Differ.* 157:109817. 10.1016/j.paid.2020.109817

[B33] Piumatti G. Salvati M. (2020). Contact with gay men and lesbian women moderates the negative relationship between religiosity and endorsement of same-sex unions’ and families’ rights. *Soc. Psychol.* 51 309–318. 10.1027/1864-9335/a000416

[B34] Puckett J. A. Woodward E. N. Mereish E. H. Pantalone D. W. (2015). Parental rejection following sexual orientation disclosure: Impact on internalized homophobia, social support, and mental health. *LGBT Health* 2 265–269. 10.1089/lgbt.2013.0024 26788675

[B35] Ren Z. J. Howe C. Q. Zhang W. (2018). Maintaining “mianzi” and “lizi”: Understanding the reasons for formality marriages between gay men and lesbians in China. *Transcult. Psychiatry* 56 213–232. 10.1177/1363461518799517 30196765

[B36] Ruan F. Fu G. Zhou M. Luo L. Chen J. Hua W. (2019). Application of the Chinese version of Zelaya’s HIV-related stigma scale to undergraduates in mainland China. *BMC Public Health* 19:1708. 10.1186/s12889-019-8054-9 31856788 PMC6923913

[B37] Sakalli N. (2002). Application of the attribution-value model of prejudice to homosexuality. *J. Soc. Psychol.* 142 264–271. 10.1080/00224540209603899 11999876

[B38] Salvati M. De Cristofaro V. Fasoli F. Paolini D. Zotti D. (2020). Introduction to the special issue: Sexual prejudice and stereotyping in modern societies. *Psicol. Soc.* 15 5–14.

[B39] Salvati M. Passarelli M. Chiorri C. Baiocco R. Giacomantonio M. (2021). Masculinity threat and implicit associations with feminine gay men: Sexual orientation, sexual stigma, and traditional masculinity. *Psychol. Men Masculin.* 22 649–668. 10.1037/men0000338

[B40] Salvati M. Pistella J. Giacomantonio M. Baiocco R. (2018). Lesbians’ negative affect toward sexual minority people with stereotypical masculine and feminine characteristics. *Int. J. Sex. Health* 30 162–176. 10.1080/19317611.2018.1472705

[B41] Salvati M. Piumatti G. Giacomantonio M. Baiocco R. (2019). Gender stereotypes and contact with gay men and lesbians: The mediational role of sexism and homonegativity. *J. Community Appl. Soc. Psychol.* 29 461–473. 10.1002/casp.2412

[B42] Song Z. Ma J. Li C. Cui Z. (2013). Development for the scale of cognition and attitudes toward gay men among medical students. *Chin. J. Sch. Health* 34 30–33.

[B43] Song Z. Ma J. Li C. Cui Z. (2014). Application of structural equation model in the analysis of the knowledge and attitude toward gay men among heterosexual medical students. *Chin. J. Health Stat.* 31 186–189.

[B44] Sousa V. D. Ryan-Wenger N. A. Driessnack D. Jaber A. F. (2010). Factorial structure of the perception of risk factors for type 2 diabetes scale: Exploratory and confirmatory factor analyses. *J. Eval. Clin. Pract.* 16 1096–1102. 10.1111/j.1365-2753.2009.01276.x 20807299

[B45] Vaughn A. A. Teeters S. A. Sadler M. S. Cronan S. B. (2016). Stereotypes, emotions, and behaviors toward lesbians, gay men, bisexual women, and bisexual men. *J. Homosex.* 64 1890–1911. 10.1080/00918369.2016.1273718 27982743

[B46] Vecho O. Gross M. Gratton E. D’amore S. Green R. J. (2019). Attitudes toward same- sex marriage and parenting, ideologies, and social contacts: The mediation role of sexual prejudice moderated by gender. *Sex. Res. Soc. Policy* 16 44–57. 10.1007/s13178-018-0331-3

[B47] Wang Y. Xin Y. Wei B. Wang J. Wang M. (2020). Influence of non-support environment, sexual identity, secret concealment on mental health of sex minority college students. *Chin. J. Health Psychol.* 23 1572–1576.

[B48] Whitley B. E. Ægisdóttir S. (2000). The gender belief system, authoritarianism, social dominance orientation, and heterosexuals’ attitudes toward lesbians and gay men. *Sex Roles* 42 947–967.

[B49] Yang X. (2020). The estimation on LGBT population size: The international experiences and China’s challenges. *Chin. J. Hum. Sex.* 29 148–152.

[B50] Yu Y. Xiao S. Xiang Y. (2010). Theory construction of the attitudes toward gay men and lesbians in Chinese context and assessments in college students. *Chin. J. Clin. Psychol.* 18 174–176. 10.16128/j.cnki.1005-3611.2010.02.024

[B51] Yu Y. Xiao S. Xiang Y. (2011). Application and testing the reliability and validity of a modified version of Herek’s attitudes toward lesbians and gay men scale in China. *J. Homosex.* 58 263–274. 10.1080/00918369.2011.540182 21294029 PMC3662080

[B52] Zhang G. Zhou H. Jing X. Chen J. Li C. Cui Z. (2016). Medical students’ attitudes towards MSM same-sex love analysis and its related factors. *Chin. J. Sch. Health* 37 197–200, 204.

[B53] Zheng Y. Xi G. Min L. (2016). Comparison of discrimination against people living with HIV/AIDS among college students between medical and normal universities. *Chin. J. AIDS STD* 22 994–995, 1004.

